# Study of gadolinium substitution effects in hexagonal yttrium manganite YMnO_3_

**DOI:** 10.1038/s41598-021-82621-6

**Published:** 2021-02-03

**Authors:** Dovydas Karoblis, Aleksej Zarkov, Edita Garskaite, Kestutis Mazeika, Dalis Baltrunas, Gediminas Niaura, Aldona Beganskiene, Aivaras Kareiva

**Affiliations:** 1 Institute of Chemistry, Vilnius University, Naugarduko 24, 03225 Vilnius, Lithuania; 2 Wood Science and Engineering, Department of Engineering Sciences and Mathematics, Luleå University of Technology, Forskargatan 1, 931 87 Skellefteå, Sweden; 3 Center for Physical Sciences and Technology, 02300 Vilnius, Lithuania; 4 Department of Organic Chemistry, Center for Physical Sciences and Technology, Sauletekio Ave. 3, 10257 Vilnius, Lithuania; 5 Institute of Chemical Physics, Faculty of Physics, Vilnius University, Sauletekio Ave. 3, 10257 Vilnius, Lithuania

**Keywords:** Inorganic chemistry, Materials chemistry, Chemical synthesis

## Abstract

In the present work, gadolinium substitution effects on the properties of yttrium manganite Y_x_Gd_1−x_Mn_0.97_Fe_0.03_O_3_ (x from 0 to 1 with a step of 0.2) synthesized by an aqueous sol–gel method have been investigated. Partial substitution of Mn^3+^ by ^57^Fe^3+^ in the manganite was also performed in order to investigate deeper the structural properties of synthesized compounds applying Mössbauer spectroscopy. It was demonstrated that substitution of Y^3+^ by Gd^3+^ ions leads to the changes of structural, magnetic and morphological properties of investigated system. The crystal structure gradually transformed from hexagonal to orthorhombic with an increase of Gd^3+^ content in the crystal lattice. The mixed phase was obtained when x = 0.6, whereas other compounds were determined to be monophasic. Magnetization measurements revealed paramagnetic behavior of all specimens, however magnetization values were found to be dependent on chemical composition of the samples. Solid solutions with orthorhombic structure revealed higher magnetization values compared to those of hexagonal samples. The highest magnetization was observed for pure GdMn_0.97_Fe_0.03_O_3_. Structural properties were investigated by powder X-ray diffraction, Mössbauer, FTIR and Raman spectroscopies. Morphological features of the synthesized specimens were studied by scanning electron microscopy (SEM).

## Introduction

Perovskite-type lanthanide manganites LnMnO_3_ (Ln—lanthanide element) have attracted a lot of interest due to their unique properties including colossal magnetoresistance and multiferroicity^1,2^. These compounds could be used as microwave phase shifters, cooling materials as well as applied in photovoltaic solar cells^3^. Depending on lanthanide element, there are two possible perovskite structures: noncentrosymmetric hexagonal (for Ln = Ho-Lu) and orthorhombic (for Ln = La-Dy)^4^. Under certain temperature and pressure one structure can be transformed to another and vice versa^5,6^. Those two perovskite phases demonstrate different magnetic configurations and electric polarization, which is higher in hexagonal structure^7^.

YMnO_3_ can be obtained in both crystal structures: thermodynamically stable hexagonal and metastable orthorhombic structure. Hexagonal YMnO_3_ is known as a single-phase multiferroic material with room-temperature ferroelectricity (T_c_ ≈ 900 K) and low-temperature antiferromagnetism (T_N_ ≈ 70 K)^8,9^. Orthorhombic YMnO_3_ is also considered as multiferroic at low temperatures, however it is characterized by lower electric polarization^10^. In addition to ferroelectric and antiferromagnetic properties, hexagonal YMnO_3_ is a narrow-band gap semiconductor, which can be utilized as photocatalyst for the degradation of organic pollutants under ultraviolet and visible light irradiation and gas sensing application^11–13^. According to literature, YMnO_3_ with orthorhombic structure can be prepared by a number of synthetic approaches such as mechanochemical synthesis^14^, epitaxial strain^15^ or other low-temperature syntheses^16^. For the preparation of hexagonal YMnO_3_ few synthesis methods were also reported, including sol–gel^17^, solid-state^18^, hydrothermal^19^, polymerized complex method^20^ and glycine-nitrate combustion^21^.

Orthorhombic GdMnO_3_ is another multiferroic rare-earth manganite, which shows few temperature-dependent magnetic transitions (from paramagnetic to antiferromagnetic collinear at 44 K, changing to A type antiferromagnetic at 23 K and weak ferromagnetism below 6 K)^22^ and large spontaneous polarization Pa ~ 4900 μC/m^2^ for thin films^23^. Hexagonal phase of this compound can be stabilized only in the form of epitaxial thin film, which was characterized by enhanced ferromagnetic properties^24^. Furthermore, this compound has high magnetoelectric coefficient and possesses pyroelectric properties^25,26^. For the preparation of GdMnO_3_ compound sol–gel^27^, solid-state^28^, sol–gel combustion^29^ and co-precipitation^30^ methods were previously applied.

There are few studies considering synthesis and characterization of Y_x_Gd_1−x_MnO_3_ system^31–35^. Vilarinho et al.^33^ and Bos et al.^35^ demonstrated the formation of Y_x_Gd_1−x_MnO_3_ solid solutions in a whole compositional range, while others investigated dielectric, magnetic and ferroelectric properties of Gd-rich orthorhombic compounds with x ≤ 0.4^31,32,34^. It was observed that partial substitution of Gd^3+^ by Y^3+^ can lead to the stabilization of ferroelectric phase when x = 0.1. Furthermore, when x > 0.1 weak ferromagnetic character disappears and antiferromagnetic ordering is established^34^. The monophasic Y_x_Gd_1−x_MnO_3_ and related systems could be employed to study and understand the charge transport across the interfaces, to investigate deeper spin-disorder state near nonmagnetic impurities, to determine substitutional–driven structure-ferroelectricity relationship and other fundamental physical properties of such complex oxides. These new knowledges obtained could be used in future for the construction of specific functionalities in novel ferroelectrics.

In this work, the solid solutions of Y_x_Gd_1−x_Mn_0.97_Fe_0.03_O_3_ (x = 0–1 with a step of 0.2) with partial substitution of Mn^3+^ by Fe^3+^ (or by ^57^Fe^3+^) ions were prepared for the first time by our best knowledge using environmentally friendly and simple sol–gel technique. Partial substitution of Mn^3+^ by ^57^Fe^3+^ in the manganites was performed in order to investigate deeper the structural and magnetic properties of synthesized compounds applying Mössbauer spectroscopy. The dependence and evolution of structural, magnetic and morphological properties on chemical composition were investigated and discussed herein.

## Materials and methods

### Synthesis

Synthesis of all samples was performed by sol–gel method using modified previously reported procedure^36^. For the preparation of Y_x_Gd_1−x_Mn_0.97_Fe_0.03_O_3_ series by changing x from 0 to 1 with a step of 0.2, yttrium (III) nitrate hexahydrate (Y(NO_3_)_3_·6H_2_O, Sigma-Aldrich, 99.9%), gadolinium (III) nitrate hexahydrate (Gd(NO_3_)_3_·6H_2_O, Sigma-Aldrich, 99.99%), manganese (II) nitrate tetrahydrate (Mn(NO_3_)_2_·4H_2_O, Alfa Aesar, 99.9%) and iron powders (Fe, Carl Roth, 99.5%) were used as the starting materials. Firstly, iron powders were dissolved in 6 M nitric acid (HNO_3_, Carl Roth, 65%) and citric acid monohydrate (C_6_H_8_O_7_·H_2_O, Chempur, 99.9%) was separately dissolved in 20 ml of distilled water. After the dissolution of citric acid, all metal nitrates and required aliquot of iron solution were added. Next, the obtained mixture was heated on a hot plate at 90 °C under constant stirring until a clear and transparent solution was obtained. After it, an appropriate amount of ethylene glycol (C_2_H_6_O_2_, Sigma-Aldrich, ≥ 99.5%) was added to the above solution (total metal ions to citric acid to ethylene glycol molar ratio was 1:3:10). The obtained liquid precursor was homogenized under constant stirring at 90 °C for 1.5 h. For the formation of the gel the temperature of magnetic stirrer was increased to 150 °C, which led to the evaporation of water. The resulted gel was dried in the oven at 140 °C for 12 h, ground in agate mortar and annealed at 1100 °C for 5 h in air with a heating rate of 5 °C/min. Identical synthesis procedure was applied for the preparation of Y_x_Gd_1−x_Mn_0.97_Fe_0.03_O_3_ (x = 0.0, 0.4 and 1.0) samples with ^57^Fe. These specimens were used only for Mössbauer spectroscopy measurements.

### Characterization

Thermal decomposition of precursor gels was investigated by thermogravimetric and differential scanning calorimetric (TG/DTG/DSC) analysis using PerkinElmer STA 6000 Simultaneous Thermal Analyzer. About 5–10 mg of dried sample was heated from 30 °C to 900 °C at 10 °C/min heating rate in a dry flowing air (20 mL/min). X-ray diffraction (XRD) analysis was performed with Rigaku Miniflex II diffractometer using a primary beam Cu Kα radiation (λ = 1.541838 Å) in 2θ range from 10° to 70° with a step of 0.02° and scanning speed of 2°/min. The obtained diffraction data were refined by the Rietveld method using the Fullprof suite. PerkinElmer FT-IR spectrometer was used for FT-IR analysis of compounds. All spectra were recorded at ambient temperature in the range of 4000–400 cm^−1^. Raman spectra were recorded using inVia Raman (Renishaw, United Kingdom) spectrometer equipped with thermoelectrically cooled (− 70 °C) CCD camera and microscope. Raman spectra were excited with 532 nm beam from the CW diode pumped solid state (DPSS) laser (Renishaw, UK). The laser power at the sample was restricted to 0.6 mW to avoid laser-induced sample heating and photodegradation. The 20×/0.40 NA objective was used during all the measurements. The overall integration time was 400 s. Position of the Raman bands on the wavenumber axis was calibrated by the polystyrene film standard spectrum. Parameters of the bands were determined by fitting the experimental spectra with Gaussian–Lorentzian shape components using GRAMS/Al 8.0 (Thermo Scientific, USA) software. The morphology of samples was investigated using a scanning electron microscope (SEM) Hitachi SU-70. Grain size distribution was estimated from SEM micrographs using ImageJ software. Magnetometer consisting of the lock-in amplifier SR510 (Stanford Research Systems), the gauss/teslameter FH-54 (Magnet Physics) and the laboratory magnet supplied by the power source SM 330-AR-22 (Delta Elektronika) was used to record magnetization dependences on applied magnetic field. Mössbauer spectra were measured using ^57^Co(Rh) source and Mössbauer spectrometer (Wissenschaftliche Elektronik GmbH) at room (≈296 K) temperature and within 10–70 K temperature range. Closed cycle He cryostat (Advanced Research Systems) was applied for low temperature measurements. The doublets, sextets, quadrupole splitting and hyperfine field distributions, and Hamiltonian method were used to fit to Mössbauer spectra applying WinNormos Site and Dist software. Combined quadrupole and magnetic dipole interactions were described using Hamiltonian method applying the parameters: hyperfine field *B*, term (main component) of quadrupole interaction ${eQ{V_{zz}}/{2}}$, asymmetry parameter *η*, and the angles *θ* and *φ*.* Q* is nuclear quadrupole moment and *V*_*zz*_ is *z* component of electric field gradient (EFG) diagonalized tensor choosing the principal axis system so that ${\left| {{V_{zz}}} \right| \geqslant \left| {{V_{xx}}} \right| \geqslant \left| {{V_{yy}}} \right|}$^37^. The asymmetry parameter ${\eta = \left( {{V_{xx}} - {V_{yy}}} \right)/{V_{zz}}}$. The angle *θ* is between magnetization direction and EFG *z* axis while the angle *φ* is between magnetization projection into the *xy* plane and EFG *x* axis. In case of pure quadrupole or magnetic dipole interactions the parameters of doublets and sextets, respectively, are determined by simple solution of Hamiltonian. The quadrupole splitting of doublet which is observed in paramagnetic state when *B* = 0 is defined by:

1 $${\Delta = \frac{{eQ{V_{zz}}}}{{2}}{{\left( {{1} + {{\frac{\eta }{{3}}}^{2}}} \right)}^{{1}/{2}}}}$$

When the quadrupole shifts *ε* of sextets lines defined by hyperfine magnetic field *B* are small they can be approximated by first order correction:

2 $${2\varepsilon = \frac{{eQ{V_{zz}}}}{{4}}\left( {{\text{3co}}{{\text{s}}^{2}}\theta - {1} + \eta {\text{si}}{{\text{n}}^{2}}\theta {\text{cos2}}\varphi } \right)}$$

The outer lines of sextet shift by + *ε*, i.e. in different direction than inner four lines shifting by –*ε*.

The influence of quadrupole and magnetic dipole interactions was of comparable strength in case of YMn_0.97_Fe_0.03_O_3_. Therefore, direct solution Hamiltonian was used for YMn_0.97_Fe_0.03_O_3_ at low temperature. For Mössbauer measurements 3 mol% of Mn was substituted by Fe (90% enriched with ^57^Fe). Because of exchange interactions iron atoms generally reflect magnetic ordering and dynamics of Mn spins in studied manganites^38–43^.

## Results and discussion

**Figure 1:**
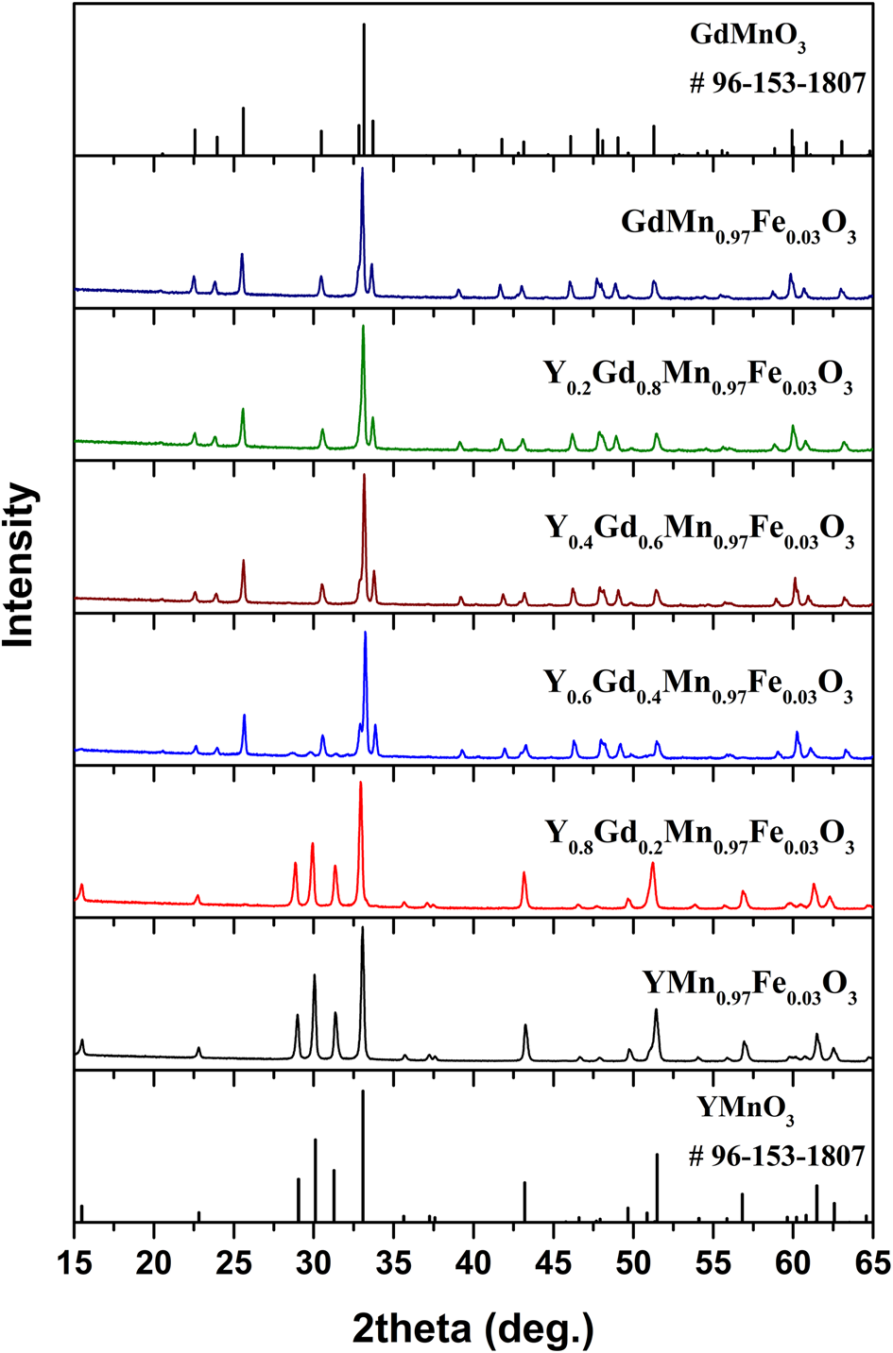
XRD patterns of Y_x_Gd_1−x_Mn_0.97_Fe_0.03_O_3_ samples.

The XRD patterns of Y_x_Gd_1−x_Mn_0.97_Fe_0.03_O_3_ samples annealed at 1100 °C are represented in Fig. 1. As was mentioned previously, few possible structures can be observed for YMnO_3_. In our case, high annealing temperature resulted in the formation of hexagonal YMn_0.97_Fe_0.03_O_3_ with P6_3_cm space group (#185). All diffraction peaks match very well with standard XRD data of hexagonal YMnO_3_ (COD #96-153-3979). The same structure was observed for x = 0.8 sample, only with a slight shift of the peaks to lower 2θ values due to the difference in the ionic radii of Gd^3+^ and Y^3+^ (ionic radius of Gd^3+^ in VII-fold coordination is 1.0 Å and for Y^3+^–0.96 Å)^44^. There was no mixture of hexagonal and orthorhombic structures observed as was suggested before for this composition^35^. With increasing the amount of Gd^3+^ the phase transition from hexagonal to orthorhombic structure can be clearly seen. In the XRD pattern of x = 0.6 sample the diffraction peaks belonging to both orthorhombic and hexagonal structures were detected. Employing Rietveld refinement for this sample, the ratio between these structures was calculated to be around 1 to 9 (10.2%—hexagonal phase, 89.8%—orthorhombic phase). These results show a significant shift towards orthorhombic structure in comparison with previous study. In^33^ the authors reported on the coexistence of nearly equal amounts of hexagonal and orthorhombic phases in Y_0.6_Gd_0.4_MnO_3_ ceramics sintered at 1350 ºC. The structure of the samples with x = 0–0.4 was determined as orthorhombic with Pnma space group (#62). Similarly, the increase in Gd^3+^ content caused the slight shift of the peaks to lower 2θ values. The narrow 2θ ranges of the XRD patterns showing gradual shift of the most intense diffraction peaks depending on chemical composition of synthesized powders are given in Figures S2 and S3. The specimens with ^57^Fe isotope (Y_x_Gd_1−x_Mn_0.97_^57^Fe_0.03_O_3_) demonstrated the identical structures (see Figure S4). No secondary phases were identified in the XRD patterns of all synthesized samples.

Rietveld refinement was performed for all synthesized samples. Calculated cell parameters and cell volumes are summarized in Table 1 and Table S1. It is seen that replacement of Y^3+^ by Gd^3+^ leads to the increase of unit cell parameters in the whole compositional range. Nearly linear dependence between chemical composition and cell parameters can be observed. The cell volume also increased with an increase of Gd^3+^ content. On the other hand, only minimal increase of *c* parameter in hexagonal structure can be seen.

**Table 1:** Cell parameters of Y_x_Gd_1−x_Mn_0.97_Fe_0.03_O_3_ samples.

Sample	Lattice parameters (Å)			
	*a*	*b*	*c*	*V*
YMn_0.97_Fe_0.03_O_3_	6.1438(4)	6.1438(4)	11.3724(0)	371.76(0)
Y_0.8_Gd_0.2_Mn_0.97_Fe_0.03_O_3_	6.1666(7)	6.1666(7)	11.3728(8)	374.54(3)
h-Y_0.6_Gd_0.4_Mn_0.97_Fe_0.03_O_3_	6.1813(5)	6.1813(5)	11.3732(0)	375.34(8)
o-Y_0.6_Gd_0.4_Mn_0.97_Fe_0.03_O_3_	5.8266(8)	7.3934(6)	5.2764(1)	227.30(4)
Y_0.4_Gd_0.6_Mn_0.97_Fe_0.03_O_3_	5.8285(5)	7.4035(0)	5.2846(7)	228.04(2)
Y_0.2_Gd_0.8_Mn_0.97_Fe_0.03_O_3_	5.8307(7)	7.4333(5)	5.3030(2)	229.84(4)
GdMn_0.97_Fe_0.03_O_3_	5.8375(4)	7.4384(8)	5.3137(5)	231.13(1)

**Figure 2:**
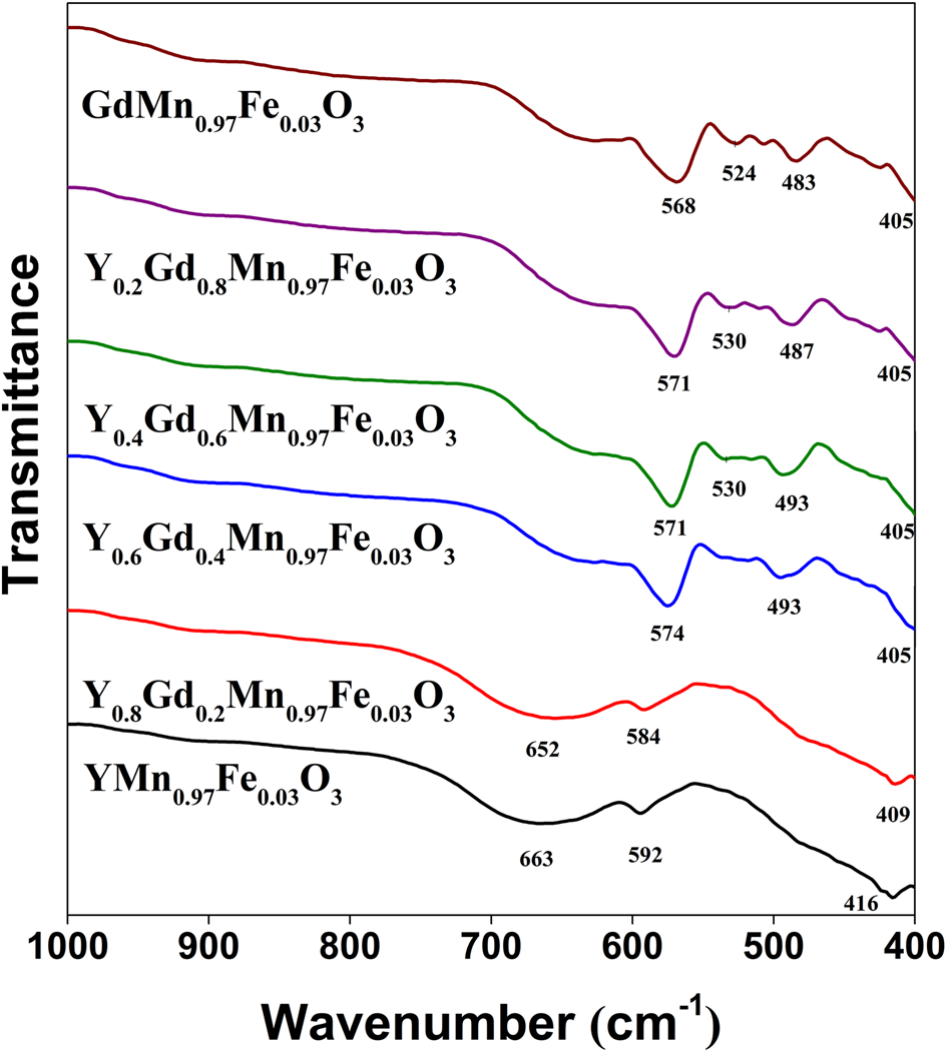
FT-IR spectra of Y_x_Gd_1−x_Mn_0.97_Fe_0.03_O_3_ samples.

Figure 2 represents FT-IR spectra of Y_x_Gd_1−x_Mn_0.97_Fe_0.03_O_3_ specimens. Three absorption bands can be observed for hexagonal samples (x = 1 and 0.8). The broad bands centered at 663 cm^−1^ (x = 1) and 652 cm^−1^ (x = 0.8) can be associated with stretching mode of Mn–O bond. This signal can be observed for all samples in the spectral range of 663–635 cm^−1^. The low intensity peaks at 592 and 584 cm^−1^ can also be attributed to the stretching vibration of Mn–O bond. Lastly, the peaks at 416 and 415 cm^−1^ can be associated with vibrations of Y–O bond, which were previously reported in^45^. Introduction of Gd^3+^ ions into the YMn_0.97_Fe_0.03_O_3_ structure obviously caused some changes in FT-IR spectra of the samples. Firstly, the sharp peak appeared in 574–568 cm^−1^ range can be clearly seen. Another lower intensity signal can be observed at 493–483 cm^−1^. Both of them are attributed to vibrations of Gd–O bond^46^. Furthermore, appearance of two peaks can also be seen for the samples with orthorhombic structure. One of them is centered at 405 cm^−1^ for all orthorhombic samples, it is associated with Mn–O vibrations^47^. The absorption band at 530 cm^−1^ is ascribed to O–Mn–O bending mode^48^. The observed spectral changes can be explained by the fact that in hexagonal structure the Mn^3+^ ions are located in trigonal bipyramid with the coordination number of 5, whereas in orthorhombic structure Mn^3+^ and six O^2−^ anions form octahedra (coordination number 6)^49^.

**Figure 3:**
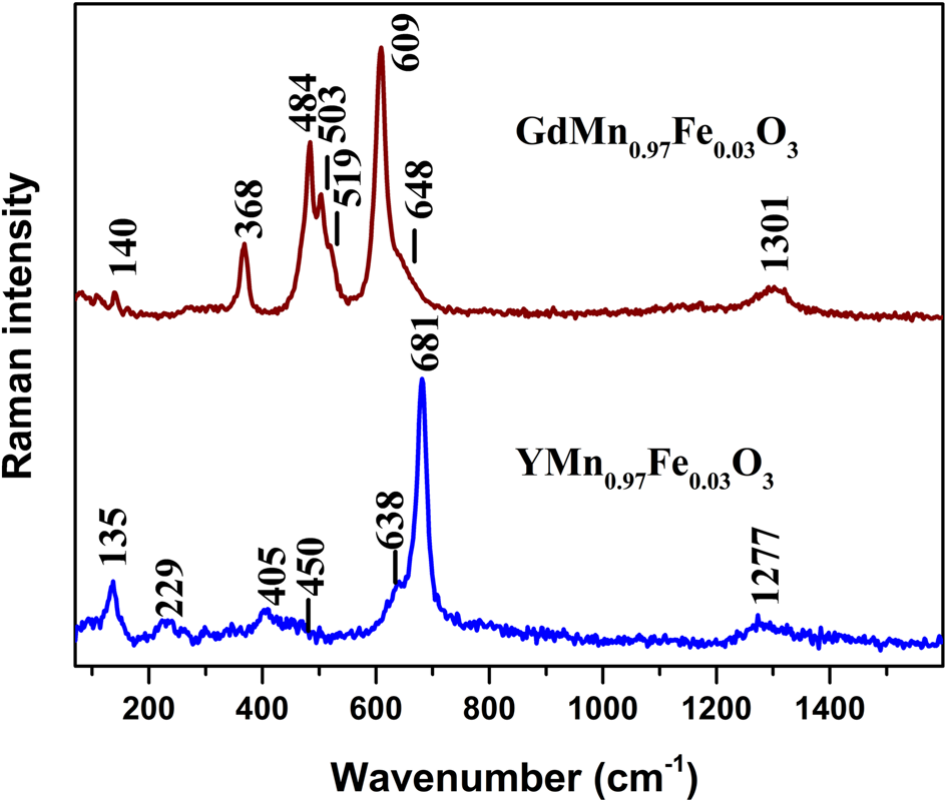
Raman spectra of polycrystalline YMn_0.97_Fe_0.03_O_3_ and GdMn_0.97_Fe_0.03_O_3_. Intensities are normalized to the intensity of the most intense band and spectra are shifted vertically for clarity. The excitation wavelength is 532 nm (0.6 mW). Parameters of the bands were determined by fitting the experimental spectra using GRAMS/Al software (version 8.0, https://www.thermofisher.com).

Raman scattering provides detailed molecular level information on short range arrangement or local symmetry which is difficult to acquire by other techniques. The method is also sensitive to structural distortions. Figure 3 compares Raman spectra of polycrystalline YMn_0.97_Fe_0.03_O_3_ and GdMn_0.97_Fe_0.03_O_3_ samples. Detailed vibrational analysis of hexagonal YMnO_3_ was performed previously by Iliev et al.^50^. The most intense band of YMn_0.97_Fe_0.03_O_3_ compound visible at 681 cm^−1^ belongs to very small or zero splitting transverse optical (TO) and longitudinal optical (LO) phonons with A_1_ symmetry considering the hexagonal structure. This mode is related to displacement of mainly oxygen atoms^50^. The shoulder at lower wavenumber side near 638 cm^−1^ is associated with TO-LO phonons of E_1_ symmetry^50^. Similarly, two low intensity bands located at 450 cm^−1^ (displacement of mainly oxygen and Mn atoms) and 405 cm^−1^ (displacement of mainly oxygen atoms) belong to A_1_ and E_1_ symmetry TO–LO modes, respectively. The low intensity band near 229 cm^−1^ can be assigned to E_2_ symmetry mode associated mainly with deformation vibration of oxygen and Mn atoms. Finally, the intense low frequency band at 135 cm^−1^ belongs to E_2_ symmetry mode related with motion of heavy Y atom. The low intensity feature near 1277 cm^−1^ is associated with overtone of oxygen stretching vibration^51^. Raman spectrum of GdMn_0.97_Fe_0.03_O_3_ differs considerably comparing with YMn_0.97_Fe_0.03_O_3_ (Fig. 3). Detailed assignments of Raman bands of orthorhombic perovskite GdMnO_3_ are provided in the publications of Illiev et al.^52^ and Oliveira et al.^53^. Thus, the most intense band at 609 cm^−1^ belongs to B_2g_(1) Jahn–Teller symmetry in-phase oxygen stretching mode. The A_g_(1) symmetry MnO_6_ bending mode is located at 503 cm^−1^. The strong band at 484 cm^−1^ is associated with A_g_(3) Jahn–Teller asymmetric stretching vibration of oxygen atoms. Finally, the well-defined band at 368 cm^−1^ belongs to A_g_(4) symmetry mode. This mode is associated with out-of-phase rotation of MnO_6_ octahedra^52^. The broad and low intensity band at 1301 cm^−1^ is related with overtone of oxygen stretching vibration^51^.

**Figure 4:**
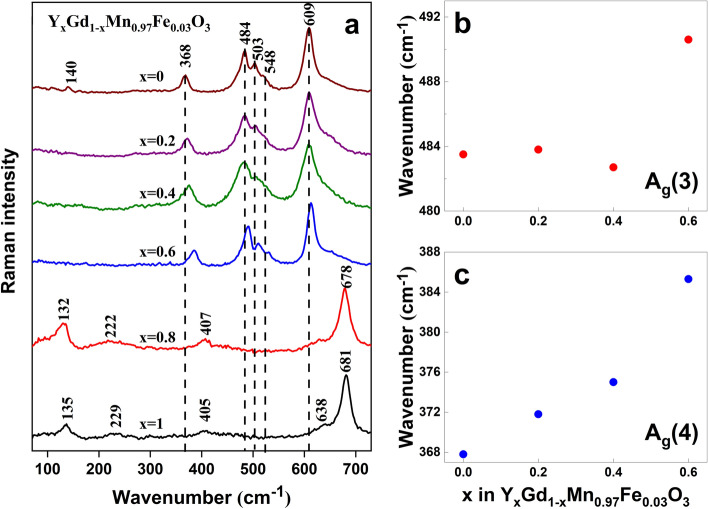
Composition dependent Raman spectra of polycrystalline Y_x_Gd_1−x_Mn_0.97_Fe_0.03_O_3_ compounds. Intensities are normalized to the intensity of the most intense band and spectra are shifted vertically for clarity. The excitation wavelength is 532 nm (0.6 mW) (**a**). Composition variation of Raman wavenumber of Y_x_Gd_1−x_Mn_0.097_Fe_0.03_O_3_ compounds for A_g_(3) mode associated with asymmetric stretching vibration of oxygens (**b**) and A_g_(4) mode associated with out-of-phase rotation of MnO_6_ octahedra (**c**). Parameters of the bands were determined by fitting the experimental spectra using GRAMS/Al software (version 8.0, https://www.thermofisher.com).

Figure 4a shows composition-dependent Raman spectra of Y_x_Gd_1−x_Mn_0.97_Fe_0.03_O_3_ compounds. The spectra corresponding to x = 1 and 0.8 are very similar indicating preservation of hexagonal YMnO_3_ structure in the case of Y_0.8_Gd_0.2_Mn_0.097_Fe_0.03_O_3_. Small distortions in the lattice are visible from the downshift of the intense A_1_ symmetry band from 681.3 to 678.7 cm^−1^, increase in relative intensities of E_1_ and E_2_ modes, and downshift of low frequency band associated with motion of Y^3+^ ion from 135.4 to 131.7 cm^−1^. Such frequency downshift correlates with larger ionic radius of Gd^3+^^44^.

However, drastic spectral changes take place after an additional introduction of Gd^3+^ to the level corresponding to Y_0.6_Gd_0.4_MnO_3_ composition. Characteristic vibrational bands of hexagonal YMnO_3_ completely disappeared. Instead, new bands characteristic to orthorhombic perovskite GdMnO_3_ appeared. Peak positions of all the observed bands downshift upon increasing the Gd^3+^ content corresponding to x = 0.6. Interestingly, further increase in Gd^3+^ amount does not affect the positions of intense bands at 609 or 484 cm^−1^ (Fig. 4b). However, different behavior was detected for the A_g_(4) mode near 368 cm^−1^ (Fig. 4c). Previously, it was demonstrated that phonon frequency of A_g_(4) mode depends linearly on the MnO_6_ hexagon rotation angle; frequency decreases with decreasing the angle^52,54^.

**Figure 5:**
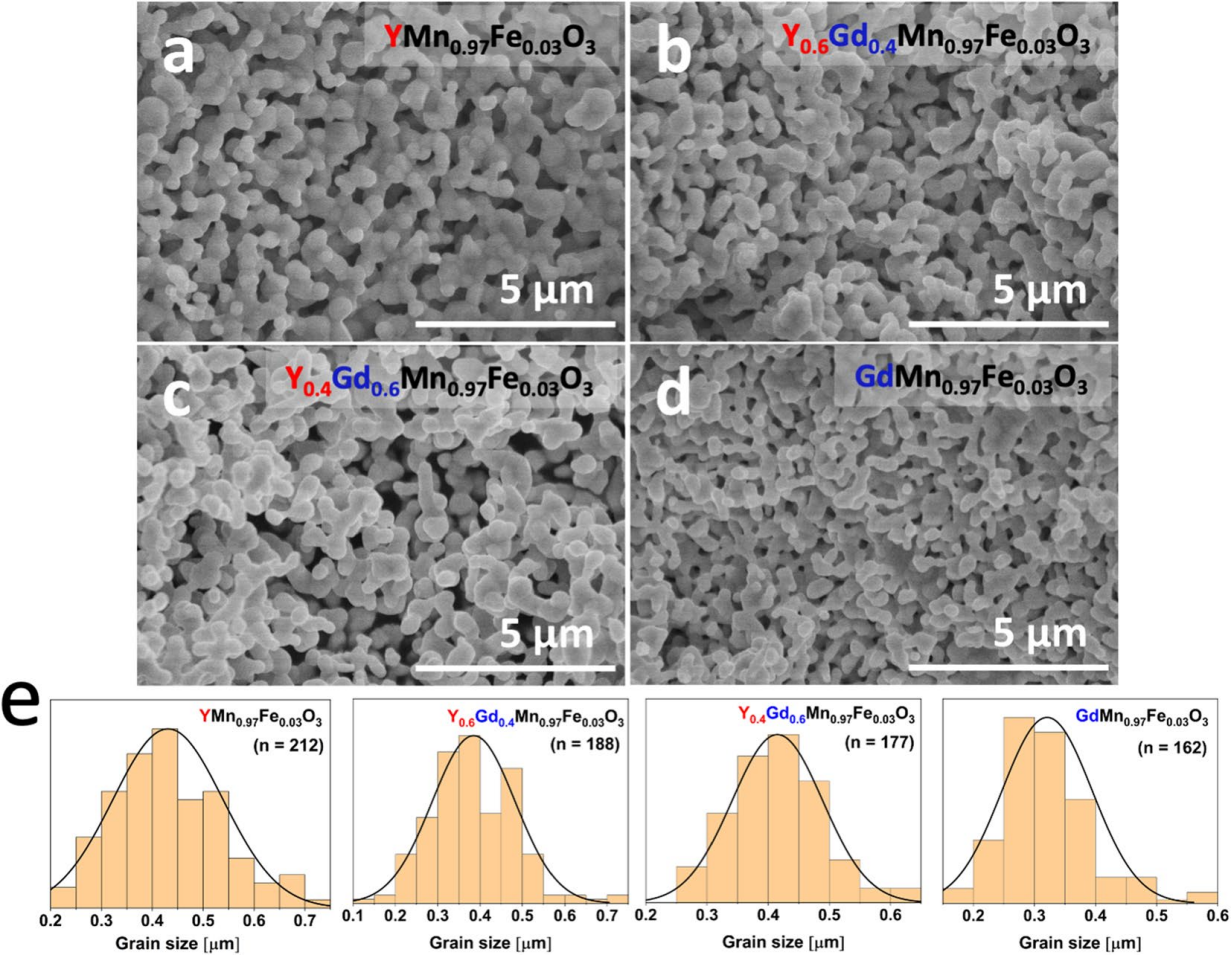
SEM micrographs of YMn_0.97_Fe_0.03_O_3_ (**a**), Y_0.6_Gd_0.4_Mn_0.97_Fe_0.03_O_3_ (**b**), Y_0.4_Gd_0.6_Mn_0.97_Fe_0.03_O_3_ (**c**) and GdMn_0.97_Fe_0.03_O_3_ (**d**) powders and histograms of grain sizes with corresponding derivatives of the cumulative distribution (**e**). Grain size distribution was calculated using ImageJ software (version 1.52 s, https://imagej.nih.gov).

Figure 5 shows SEM micrographs of Y_x_Gd_1−x_Mn_0.097_Fe_0.03_O_3_ powders annealed at 1100 ºC and histograms of grain sizes with corresponding derivatives of cumulative distribution. It can be seen that YMn_0.97_Fe_0.03_O_3_ powder (Fig. 5a) possesses porous structure and consists of sintered aggregates which are composed of smaller and mostly uniform particles necked to each other. The histogram of grain size distribution (Fig. 5e) shows that grain size varies in the range of approximately 200–700 nm (around 90% of the grains; derivative of the cumulative distribution has maximum at 433 nm). The SEM micrographs of Y_0.6_Gd_0.4_Mn_0.097_Fe_0.03_O_3_ and Y_0.4_Gd_0.6_Mn_0.097_Fe_0.03_O_3_ powders (Fig. 5b,c) show that particles having similar sizes and shape were formed, but slightly narrower size range was observed compared to the undoped sample (derivative maxima were obtained at 384 nm and 415 nm for Y_0.6_Gd_0.4_Mn_0.097_Fe_0.03_O_3_ and Y_0.4_Gd_0.6_Mn_0.097_Fe_0.03_O_3_, respectively). Porous structure was also maintained after the Gd was introduced as dopant. The different morphology was observed for the GdMn_0.97_Fe_0.03_O_3_ material (Fig. 5d). As seen, GdMn_0.97_Fe_0.03_O_3_ powder possesses the smallest grains; and the histogram shows that around 90% of all grains are distributed in 100–500 nm range and around 70% of grains lie in considerably narrower range from 200 to 400 nm (derivative maximum at 320 nm). This shows that with an increase in Gd content the average grain size of Y–Gd–Mn–Fe–O powders becomes smaller and suggests that the surface area and porosity of such ceramic materials could be tailored by changing chemical composition.

**Figure 6:**
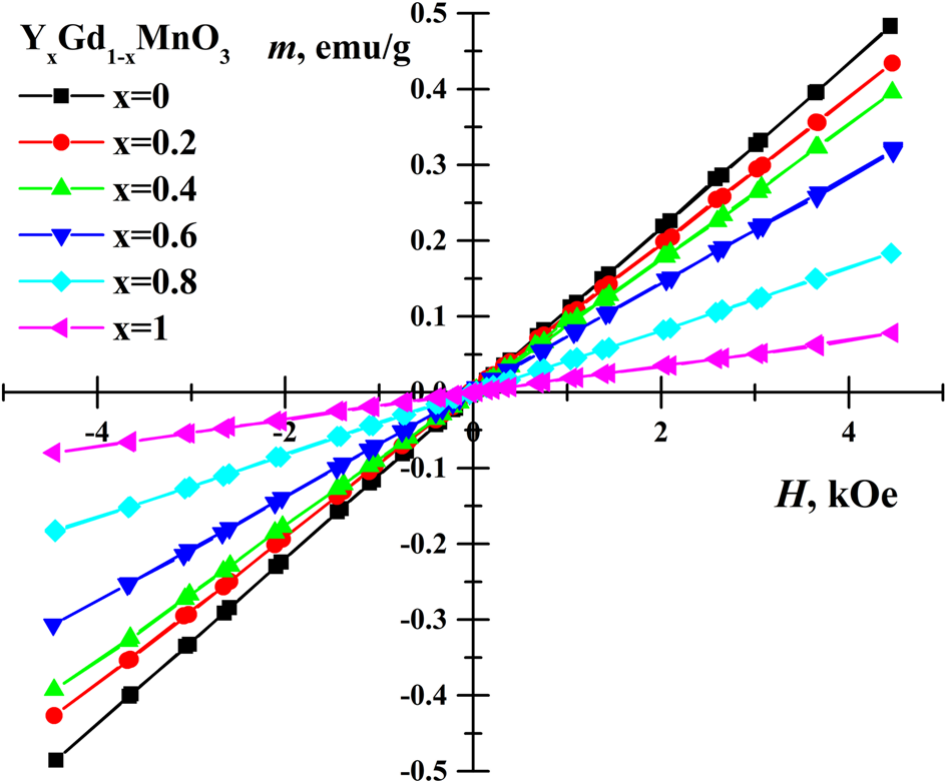
Magnetization hysteresis of Y_x_Gd_1−x_Mn_0.97_Fe_0.03_O_3_ samples at room temperature.

Dependence of the magnetization on applied magnetic field strength was studied for all samples and results are presented in Fig. 6. Linear magnetization dependences $m = \chi H$ were observed for all Y_x_Gd_1−x_Mn_0.97_Fe_0.03_O_3_ solid solutions, which corresponds to paramagnetic state of the materials. Magnetic susceptibility of rare earth manganites is due to both Gd and Mn magnetic moments. The Curie–Weiss law ${\chi_{mol}} = {{{N_A}{\mu_{eff}}^2} \mathord{\left/ {\vphantom {{{N_A}{\mu_{eff}}^2} {\left( {3{k_B}\left( {T - \theta } \right)} \right)}}} \right. \kern-\nulldelimiterspace} {\left( {3{k_B}\left( {T - \theta } \right)} \right)}}$ was used to describe dependence of magnetic (molar) susceptibility, where *N*_*A*_ and *k*_*B*_ are the Avogadro number and Boltzmann constant on temperature. Application of Curie–Weiss law gave Curie–Weiss temperature *θ* = -421 K and *θ* ≈-35 K for YMnO_3_ and GdMnO_3_^39,55^, respectively. The effective magnetic moment μ_eff_≈9.4 μ_B_ of GdMnO_3_ was considerably larger than μ_eff_ = 4.98 μ_B_ for YMnO_3_, where μ_B_ is Bohr magneton. Therefore, the inclination magnetization lines increase with amount of Gd^3+^ because of both change in *θ* and μ_eff_. It can be noticed that more significant decrease in magnetization values was observed along with transformation from orthorhombic structure to hexagonal (between x = 0.6 and 0.8).

**Figure 7:**
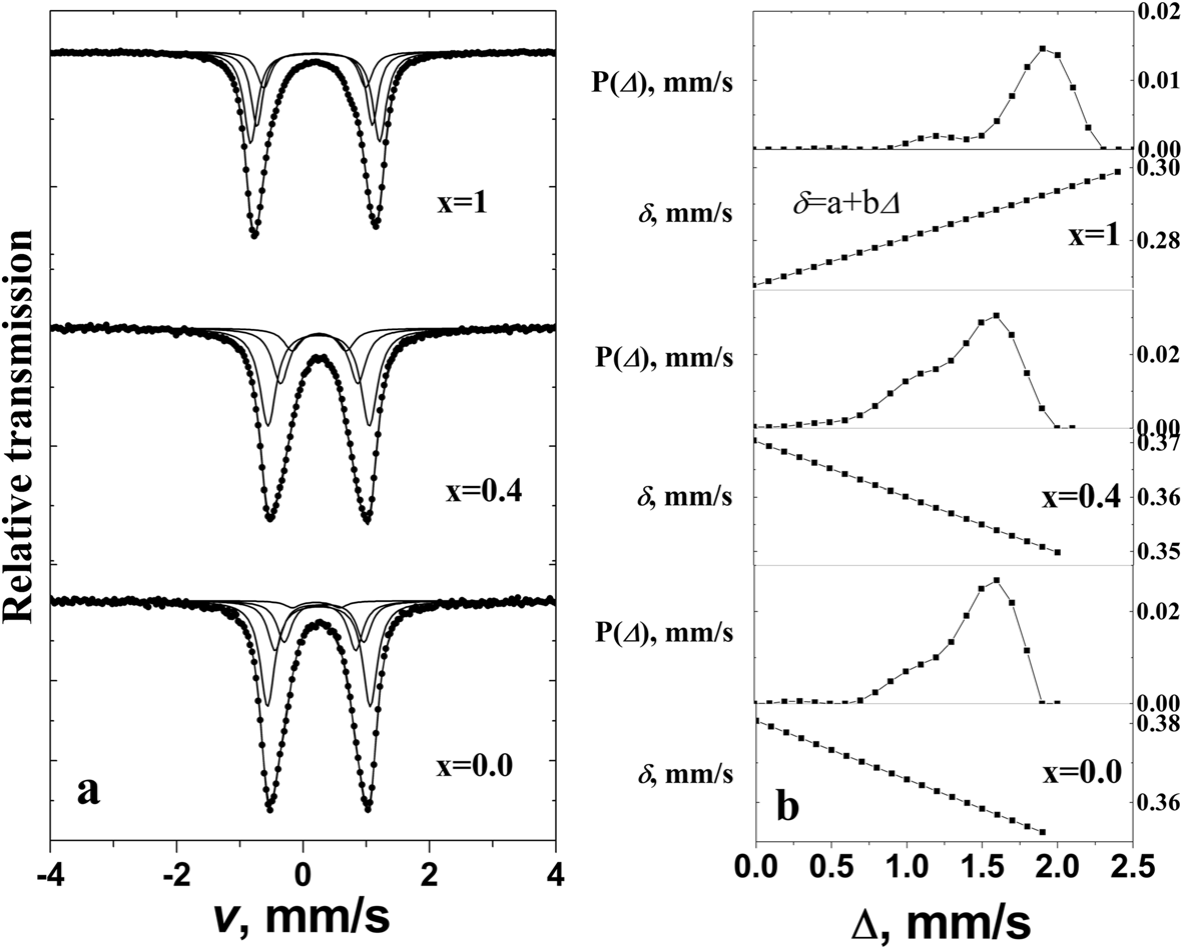
Mössbauer spectra of Y_x_Gd_1−x_Mn_0.97_Fe_0.03_O_3_ at room temperature fitted to doublets (**a**) and quadrupole splitting distribution P(*∆*) with dependence of isomer shift *δ* on quadrupole splitting *∆* (**b**). Fitting of the spectra was performed using WinNormos-for-Igor software package (version 3.0, http://www.wissel-gmbh.de).

Two alternative methods, quadrupole splitting distributions P(∆) with quadrupole splitting ∆ step of 0.1 mm/s and three or four doublets with freely variable parameters, were used for fitting to the room temperature Mössbauer spectra of Y_x_Gd_1−x_Mn_0.97_Fe_0.03_O_3_ (Fig. 7, Table 2). Quadrupole splitting distributions P(∆) have peaks approximately at 1.60, 1.58 and 1.95 mm/s for x = 0, 0.4 and 1, respectively. However, the distributions are wide; therefore, they indicate that Fe sites differ significantly by quadrupole splitting. The wider distribution of mixed Y_0.4_Gd_0.6_Mn_0.97_Fe_0.03_O_3_ in comparison to other two (Fig. 7b) can be explained by different influence of Y^3+^ and Gd^3+^ to the local crystal structure. In case of application of separate doublets, the most intense doublet has largest quadrupole splitting ∆ (Table 2) which value can be explained by significant distortions of bipyramid MnO_5_ and octahedra MnO_6_ in hexagonal and orthorhombic RMnO_3_ (R is rare earth) structures evaluating EFG components with application of point charge model^38,40,41^. The doublets of smaller splitting should be attributed to more symmetric Fe sites. According to previous Mössbauer study of hexagonal YFe_y_Mn_1−y_O_3_^38^ the relative area of doublet with smaller ∆ increased with Mn substitution by Fe. In the studies^39,42^ additional doublets at y = 0.1; 0.2 were related with Fe atoms occupying Mn sites in nearest neighborhood. However, at 3% Mn substitution the observed intensity of additional doublet was much larger than could be according to random occupation of six neighboring sites by Fe. Probably, the number of defects which increases in case of ultrafine structure, may also have similar influence. The isomer shift which is proportional to electron density at Fe nucleus^37^ depends on the Fe coordination number because of redistribution of electron charge. The isomer shift *δ*≈0.29 mm/s of hexagonal YMn_0.97_Fe_0.03_O_3_ was smaller than *δ*≈0.36 mm/s for orthorhombic phases Y_x_Gd_1−x_Mn_0.97_Fe_0.03_O_3_ with x = 0, 0.4 (Table 2). Comparing hexagonal and orthorhombic phases it can be noticed that the dependence of isomer shift *δ* on quadrupole splitting *∆* is of different sign (Fig. 7b). Moreover, it can be noted that the ratio of intensities of doublet lines A_12_ is slightly deviated from 1 (Table 2). Such effect can be caused by Goldanskii-Karyagin effect or sample anisotropy^56^.

**Table 2:** Parameters of doublets used to fit to Y_x_Gd_1−x_Mn_0.97_Fe_0.03_O_3_ Mössbauer spectra at room temperature: *S* is relative area, *Γ*—linewidth, *δ—*isomer shift relatively to α-Fe at room temperature, Δ—quadrupole splitting, *A*_*12*_*—*line area ratio.

*x*	*S*, %	*Γ*, mm/s	*δ*, mm/s	*Δ*, mm/s	A_12_
0	38	0.254 ± 0.014	0.356 ± 0.001	1.668 ± 0.013	0.952 ± 0.004
*χ*^2^ = 1.12	32	0.26 ± 0.03	0.362 ± 0.003	1.435 ± 0.017	0.952*
	30	0.345 ± 0.012	0.376 ± 0.003	1.113 ± 0.017	0.952*
	*average*		0.364 ± 0.001	1.425 ± 0.009	
0.4	34	0.27 ± 0.01	0.352 ± 0.001	1.662 ± 0.013	0.954 ± 0.003
*χ*^2^ = 1.34	29	0.30 ± 0.04	0.358 ± 0.001	1.403 ± 0.016	0.954*
	37	0.41 ± 0.01	0.370 ± 0.002	1.065 ± 0.015	0.954*
	*average*		0.361 ± 0.001	1.425 ± 0.009	
1	42	0.254 ± 0.004	0.298 ± 0.001	2.039 ± 0.008	0.983 ± 0.002
*χ*^2^ = 1.53	28	0.23 ± 0.04	0.290 ± 0.001	1.828 ± 0.011	0.983*
	18	0.26 ± 0.05	0.293 ± 0.001	1.61 ± 0.04	0.983*
	12	0.339 ± 0.015	0.282 ± 0.002	1.190 ± 0.009	0.983*
	*average*		0.292 ± 0.001	1.801 ± 0.006	

*All equal.

**Figure 8:**
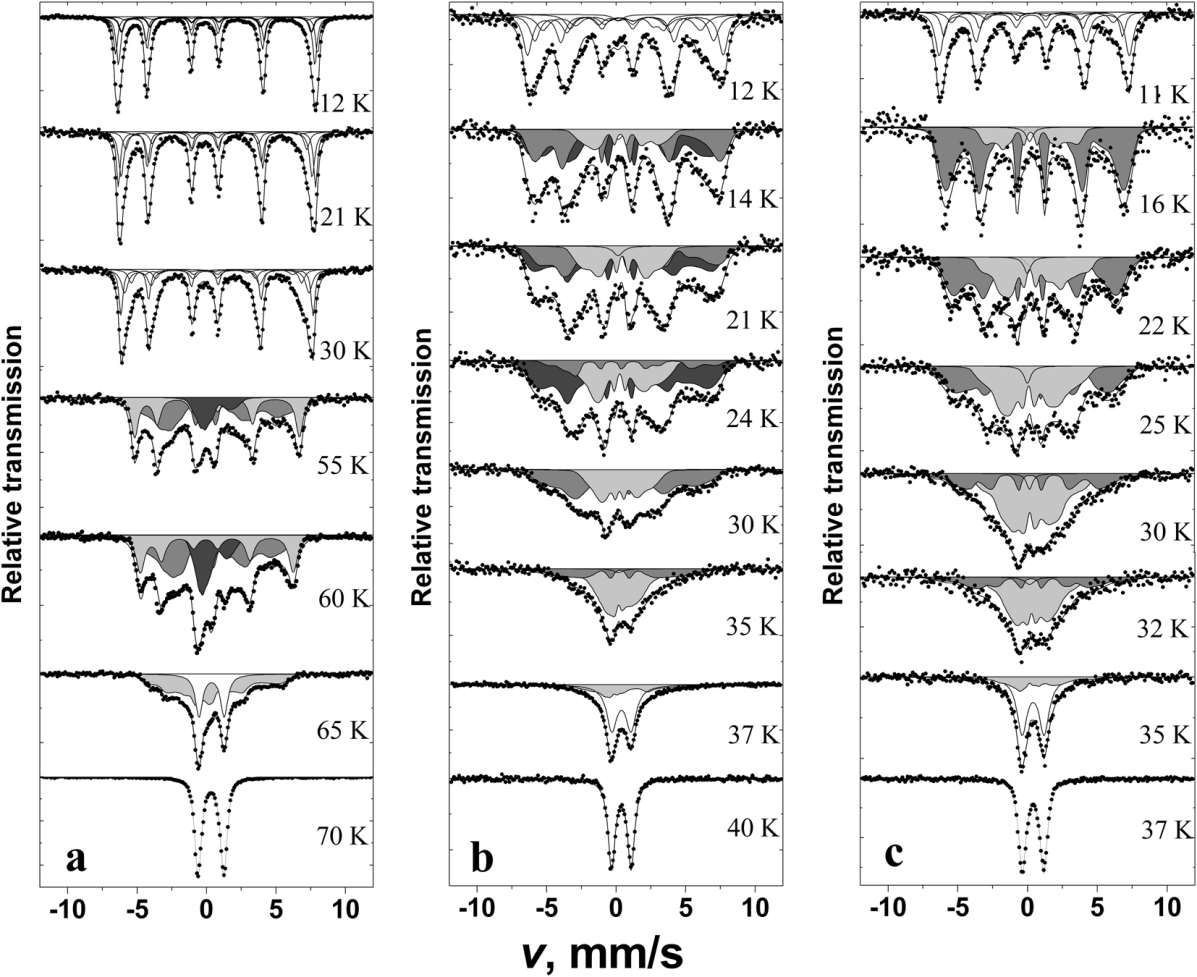
Mössbauer spectra of YMn_0.97_Fe_0.03_O_3_ (**a**), Y_0.4_Gd_0.6_ Mn_0.97_Fe_0.03_O_3_ (**b**) and GdMn_0.97_Fe_0.03_O_3_ (**c**) at indicated temperatures. Fitting of the spectra was performed using WinNormos-for-Igor software package (version 3.0, http://www.wissel-gmbh.de).

**Figure 9:**
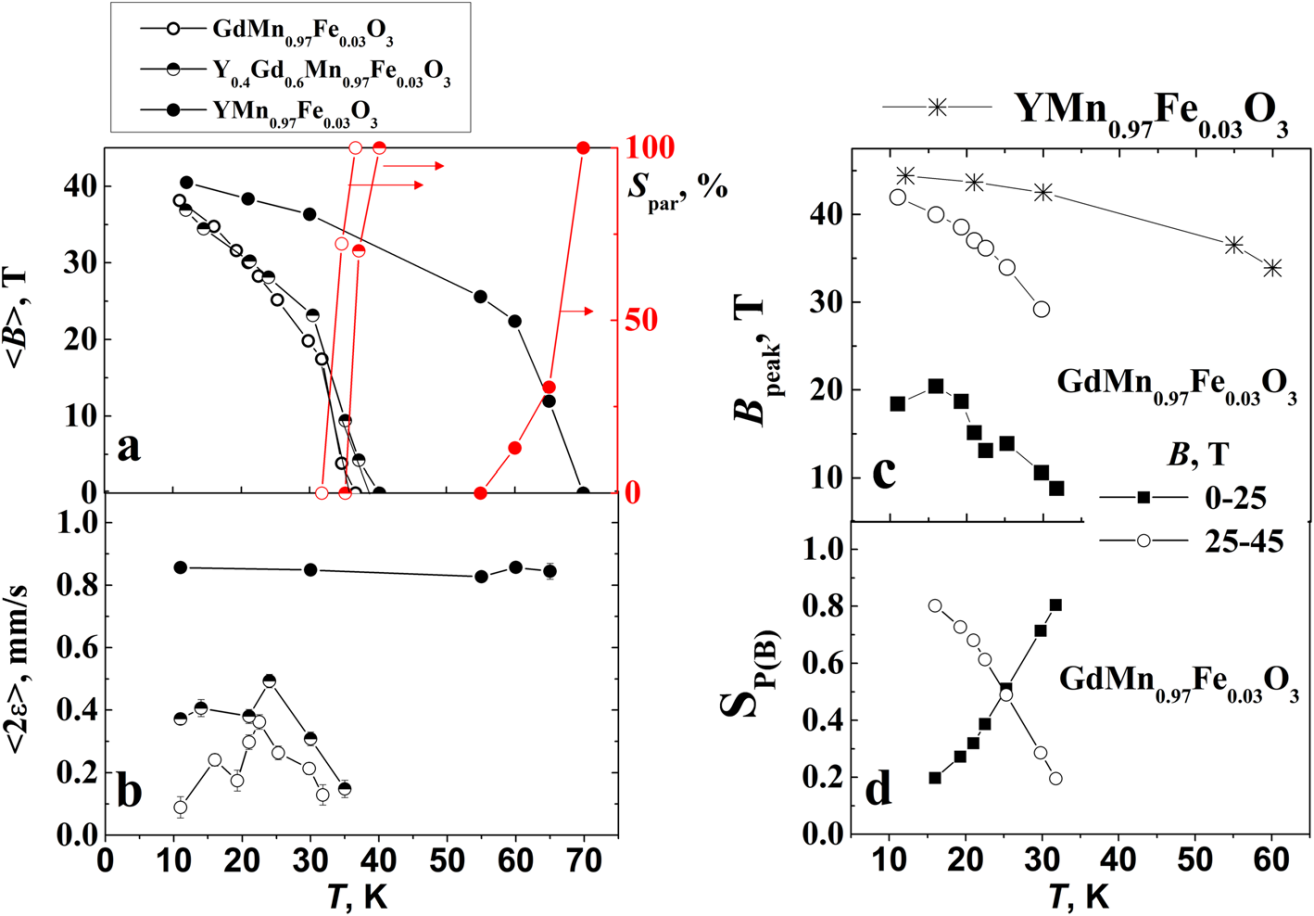
Dependence of average hyperfine field < *B* > (left), relative area of doublet S (right) (**a**), average quadrupole shift < 2ε> (**b**) for YMn_0.97_Fe_0.03_O_3_, Y_0.4_Gd_0.6_Mn_0.97_Fe_0.03_O_3_, and GdMn_0.97_Fe_0.03_O_3_ on temperature. The peaks positions (**c**) and the relative area of hyperfine field distributions P(*B*) (d) within 0–25 and 25–45 T regions on temperature.

According to low temperature Mössbauer spectra the magnetic ordering occurs at ≈ 36, 39, 70 K for Y_x_Gd_1−x_Mn_0.97_Fe_0.03_O_3_ with x = 0, 0.4 and 1, respectively (Figs. 8 and 9a). It was previously observed for YFe_y_Mn_1−y_O_3_ that increasing *y* from 0.02 to 0.2 magnetic ordering temperature decreased from 73 to 60 K^38^. Crystal structure (hexagonal or orthorhombic) of RMnO_3_ and Mn–O-Mn angle in orthorhombic RMnO_3_ affect magnetic ordering temperature according to^38–43,55,57,58^. Mössbauer spectrum of YMn_0.97_Fe_0.03_O_3_ measured at 12 K (Fig. 8a) was fitted to subspectra using Hamiltonian method as the quadrupole shifts were too large to consider only first order corrections (Eq. (2)). The term of quadrupole interaction was fixed according to room temperature data, $\left| {eQ{V_{zz}}} \right|/{2}{ \approx \Delta }$ (Eq. (1), Tables 2 and 3). In case of hexagonal YMn_0.97_Fe_0.03_O_3_ the angle *θ* = 90° (Table 3) corresponds to EFG *z* axis along crystal *c* axis and the magnetization in *ab* plane^39,42,58,59^. The angle *φ* between magnetization projection into EFG *xy* plane and *x* axis had small influence on fitting quality and was fixed to 0 or 90° trying to keep *0* < *η *< 1 (Table 3)*.* When asymmetry of EFG^37^ which expressed by parameter *η* is small, according to Eq. (2) the spins of Fe lying at different angles in *ab* plane resulted only in small changes in position of lines of Mössbauer spectrum and can be ascribed to the same subspectrum with slightly larger width of lines. Negative sign of ${eQ{V_{zz}}/{2}}$ for YMn_0.97_Fe_0.03_O_3_ was in agreement with point charge calculations of EFG in case of hexagonal structure of rare earth manganites YMnO_3_ and YbMnO_3_^38,40^. Four subspectra (Table 3) which were fitted to Mössbauer spectrum of YMn_0.97_Fe_0.03_O_3_ measured at 12 K correspond to doublets because of fixing ${eQ{V_{zz}}/{2}}$ and area ratios of subspectra. Largest difference in hyperfine field *B* values of subspectra was ≈3 T indicating that the spectrum is broadened because of variation of both dipole magnetic and quadrupole interactions.

**Table 3:** Parameters of sextets and Hamiltonian used to fit to Y_x_Gd_1−x_Mn_0.97_Fe_0.03_O_3_ Mössbauer spectra measured at 11–12 K: *S* is relative area, *Γ*–linewidth, δ—isomer shift relatively to α-Fe at room temperature, 2*ε*—quadrupole shift, ${{eQ{V_{zz}}} \mathord{\left/ {\vphantom {{eQ{V_{zz}}} 2}} \right. \kern-\nulldelimiterspace} 2}$—term of quadrupole interaction, *B*—hyperfine field, *η*—asymmetry parameter, *θ* is the angle between magnetization and EFG *z* axis and *φ *is the angle between magnetization projection and EFG *x* axis*.*

*x*	*S*, %	*Γ* mm/s	*δ*, mm/s	2*ε*, mm/s	*B*, T			
0	52	0.67 ± 0.03	0.493 ± 0.006	0.22 ± 0.01	42.33 ± 0.08	–	–	–
	15	0.48 ± 0.08	0.463 ± 0.013	0.13 ± 0.03	39.80 ± 0.13	–	–	–
	15	0.74 ± 0.10	0.50 ± 0.02	− 0.06 ± 0.05	36.0 ± 0.2	–	–	–
	18	2.1 ± 0.4	0.493*	− 0.2 ± 0.2	20.0 ± 0.8	–	–	–
0.4	34	0.75 ± 0.05	0.481 ± 0.007	0.57 ± 0.02	43.6 ± 0.1	–	–	–
	27	0.84 ± 0.09	0.481*	0.41 ± 0.03	39.9 ± 0.2	–	–	–
	26	1.04 ± 0.09	0.481*	0.04 ± 0.04	35.1 ± 0.2	–	–	–
	13	0.78 ± 0.08	0.481*	− 0.64 ± 0.05	20.9 ± 0.2	–	–	–

				${{eQ{V_{zz}}} \mathord{\left/ {\vphantom {{eQ{V_{zz}}} 2}} \right. \kern-\nulldelimiterspace} 2}$, mm/s		*η*	*θ, °*	*ϕ, °*
1	42	0.36 ± 0.01	0.413 ± 0.001	− 2.039*	42.97 ± 0.01	0.128 ± 0.003	90*	0*
	28	0.33 ± 0.01	0.418 ± 0.002	− 1.828*	44.42 ± 0.01	0.066 ± 0.004	90*	0*
	18	0.42 ± 0.01	0.423 ± 0.004	− 1.61*	41.89 ± 0.04	0.018 ± 0.009	90*	90*
	12	0.73 ± 0.03	0.405 ± 0.012	− 1.19*	40.07 ± 0.11	0.38 ± 0.04	90*	90*

*Fixed.

At lowest 11–12 K temperature the lines of Mössbauer spectra of GdMn_0.97_Fe_0.03_O_3_ and especially Y_0.4_Gd_0.6_Mn_0.97_Fe_0.03_O_3_ (Fig. 8b,c) were broader than those of YMn_0.97_Fe_0.03_O_3_. Four sextets with different parameters *B* and 2*ε* were fitted to Mössbauer spectra (Table 3). Quadrupole shifts of subspectra were smaller than those of YMn_0.97_Fe_0.03_O_3_ calculated according to Eq. (2) as shown in Fig. 9b, but hyperfine field *B* of sextets varied from 20 to ≈42 T. Such differences arise because of lower magnetic ordering temperature, crystal structure and spin ordering specifics.

**Figure 10:**
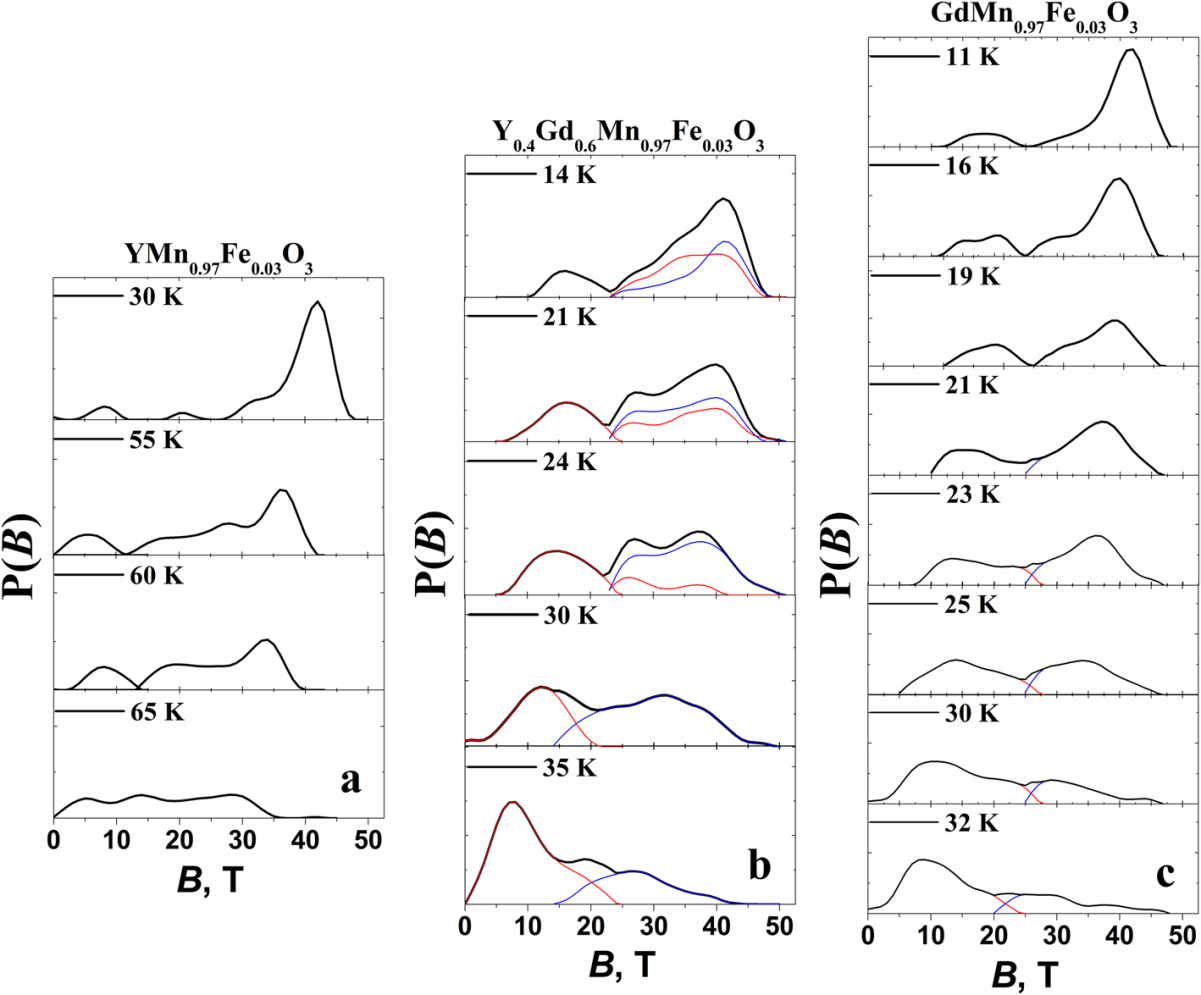
Hyperfine field distributions P(*B*) of YMn_0.97_Fe_0.03_O_3_ (**a**), Y_0.4_Gd_0.6_Mn_0.97_Fe_0.03_O_3_ (**b**), and GdMn_0.97_Fe_0.03_O_3_ (**c**). Fitting was performed using WinNormos-for-Igor software package (version 3.0, http://www.wissel-gmbh.de).

Hyperfine field distributions P(*B*) were used for fitting to the Mössbauer spectra (Figs. 8 and 10) measured at higher then 11–12 K temperature up to transition to paramagnetic state. The shape of distributions is characterized by the features that are specific of different studied samples. Hyperfine distributions of YMn_0.97_Fe_0.03_O_3_ exhibited one peak within 35–45 T hyperfine field range which intensity decreased as temperature increased (Figs. 9c and 10a). Two peaks within 30–45 and 10–20 T hyperfine field regions (Fig. 9c) were characteristic of hyperfine field distributions P(*B*) of GdMn_0.97_Fe_0.03_O_3_. Broader hyperfine distributions of Y_0.4_Gd_0.6_Mn_0.97_Fe_0.03_O_3_ were observed because of different influence of Y and Gd on Fe local surrounding.

Dividing P(*B*) of GdMn_0.97_Fe_0.03_O_3_ into two parts (hyperfine field *B* varies in the range of 0–25 and 25–45 T) it can be shown that intensities of high and low hyperfine field regions were approximately equal at 23 K (Fig. 9d) when the magnetic ordering transition in GdMnO_3_ occurs^55,57,58,60^. P(*B*) within 25–45 T region dominates at low 11–12 K temperature (Fig. 10c, Table 3) when Mn spin order in GdMnO_3_ is A type antiferromagnetic. Incommensurate collinear (IC) sinusoidal amplitude modulated spin order (spin lying along *a* axis of *Pnma* space group or *b* axis in *Pbnm*) at temperature higher than 23 K should lead to P(*B*) distribution in a wide hyperfine field range as observed for FeVO_4_ and orthorhombic Fe-doped YbMnO_3_^43,61^ with a maximum of *B*≈38 T. However, we observed the distribution of P(*B*) for GdMn_0.97_Fe_0.03_O_3_ having another peak at *B* = 10–20 T and minimum at *B* = 20–25 T which could be hardly related with IC sinusoidal amplitude modulated spin order. Certainly, Fe spin coupled by exchange interactions with nearest-neighbor Mn should be affected by Mn spin modulation. The exchange interactions of Mn–O–Mn in studied materials are ferromagnetic or antiferromagnetic depending on crystallographic directions^57^. However, the doping with Fe may lead to some changes in spin order compared to GdMnO_3_ because of the exchange interactions of Mn–O–Mn being of different strength compared to ferromagnetic Mn–O–Fe or antiferromagnetic Fe–O–Fe exchange interactions. Structural differences in Fe sites which are indicated by quadrupole splitting changes at room temperature should also affect spin order. In case of Y_0.4_Gd_0.6_Mn_0.97_Fe_0.03_O_3_ three hyperfine field distributions P(*B*) were used (Fig. 10b). Two P(*B*) having different quadrupole shift were needed in 25–45 T range, probably because of different Y and Gd influence on Fe local surrounding. In this case even more complicated spin order may exist.

Mössbauer spectra broadening and lines shift to center when temperature increases are explained by the increase in spin relaxation rate. According to Mössbauer spectra line shape theory^62,63^ the shape depends on population of stochastic spin states and transition rate between these states. Different stochastic states of Fe occur because of thermal excitations of spins, collective excitations such as magnons. At characteristic time of transition between stochastic states less than 10^–7^ s the shape of spectra starts to change (Mössbauer lines broaden) and at less than 10^–9^–10^–10^ s averaged states (merged sextet lines to doublet/singlet at paramagnetic state) are observed. The change in peak positions *B*_peak_ of GdMn_0.97_Fe_0.03_O_3_ within 25–45 and 0–25 T hyperfine field regions, when temperature increases, was rather slow similarly to that of one peak position of YMn_0.97_Fe_0.03_O_3_ in Figs. 9c and 10a. The gradual probability shift from the higher *B* region with P(*B*) peak at *B* = 30–45 T to another one with 10–20 T peak with increasing temperature is associated with the decrease in hyperfine field because of thermal excitation and averaging of states. The spin order transitions can also contribute to lowering of hyperfine field, however, we do not observe any abrupt changes at 23 K (Fig. 9a).

## Conclusions

A series of Y_x_Gd_1−x_Mn_0.97_Fe_0.03_O_3_ (x from 0 to 1 with a step of 0.2) powders has been synthesized by an aqueous sol–gel method, and gadolinium substitution effects in yttrium manganite were investigated. Partial substitution of Mn^3+^ by ^57^Fe^3+^ was performed in order to investigate deeper the structural properties of synthesized compounds applying Mössbauer spectroscopy. With increasing the Gd^3+^ content in the samples the crystal structure of Y_x_Gd_1−x_Mn_0.97_Fe_0.03_O_3_ gradually transformed from hexagonal to orthorhombic. The mixed phase was obtained when x = 0.6, whereas other compounds were determined to be monophasic. It was demonstrated that cell parameters increased almost linearly with increasing amount of Gd^3+^ in Y_x_Gd_1−x_Mn_0.97_Fe_0.03_O_3_. The results of FTIR and Raman spectroscopies were in good agreement with ones obtained by XRD analysis. According to SEM micrographs, the most of the samples were composed of porous aggregates which are organized of significantly smaller and mostly uniform particles necked to each other. The particle size varies in the range of approximately 100–600 nm depending on the chemical composition of gadolinium-substituted yttrium manganites. All synthesized compounds were characterized by paramagnetic behavior at room temperature; however, magnetization values were found to be dependent on chemical composition of the samples. Solid solutions with orthorhombic structure revealed higher magnetization values compared to those of hexagonal samples. According to Mössbauer spectroscopy data the magnetic ordering occurs at ≈ 36, 39 and 70 K for Y_x_Gd_1−x_Mn_0.97_Fe_0.03_O_3_ with x = 0, 0.4 and 1, respectively. For orthorhombic GdMn_0.97_Fe_0.03_O_3_ the change in types of antiferromagnetic ordering at 23 K is associated with the increase in hyperfine field probability of distribution within 25–45 T relatively to 10–20 T region which is more intense at higher temperature.

## Supplementary Information

Supplementary Information
