# Supplementary material for: Study of gadolinium substitution effects in hexagonal yttrium manganite YMnO3
Source: Sci Rep. 2021 Feb 3;11:2875. doi: 10.1038/s41598-021-82621-6 (PMC7859184; doi:10.1038/s41598-021-82621-6)
Supplement: Supplementary file 1 — Supplementary Information [file 41598_2021_82621_MOESM1_ESM.pdf]

Supporting Information

Study of gadolinium substitution effects in hexagonal yttrium manganite $YMnO_3$

Dovydas Karoblis[1], Aleksej Zarkov[1,*], Edita Garskaite[2], Kestutis Mazeika[3], Dalis Baltrunas[3], Gediminas Niaura[4,5], Aldona Beganskiene[1], Aivaras Kareiva[1]

[1]*Institute of Chemistry, Vilnius University, Naugarduko 24, LT-03225 Vilnius, Lithuania*

[2]*Wood Science and Engineering, Department of Engineering Sciences and Mathematics, Luleå University of Technology, Forskargatan 1, SE-931 87 Skellefteå, Sweden*

[3]*Center for Physical Sciences and Technology, Vilnius LT-02300, Lithuania*

[4]*Department of Organic Chemistry, Center for Physical Sciences and Technology, Sauletekio Ave. 3, LT-10257, Vilnius, Lithuania*

[5]*Institute of Chemical Physics, Faculty of Physics, Vilnius University, Sauletekio Ave. 3, LT-10257, Vilnius, Lithuania*

[*]*Corresponding author: E-mail: aleksej.zarkov@chf.vu.lt*

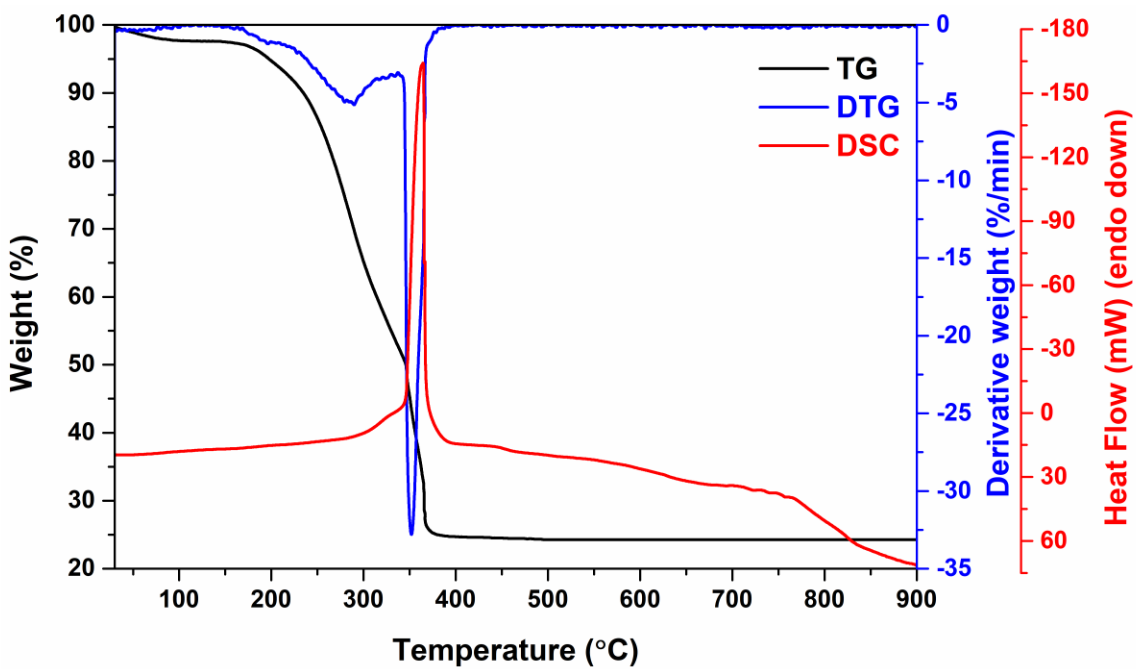

Figure S1. TG/DTG/DSC curves of Y-(0.97)Mn-(0.03)Fe-O precursor gel.

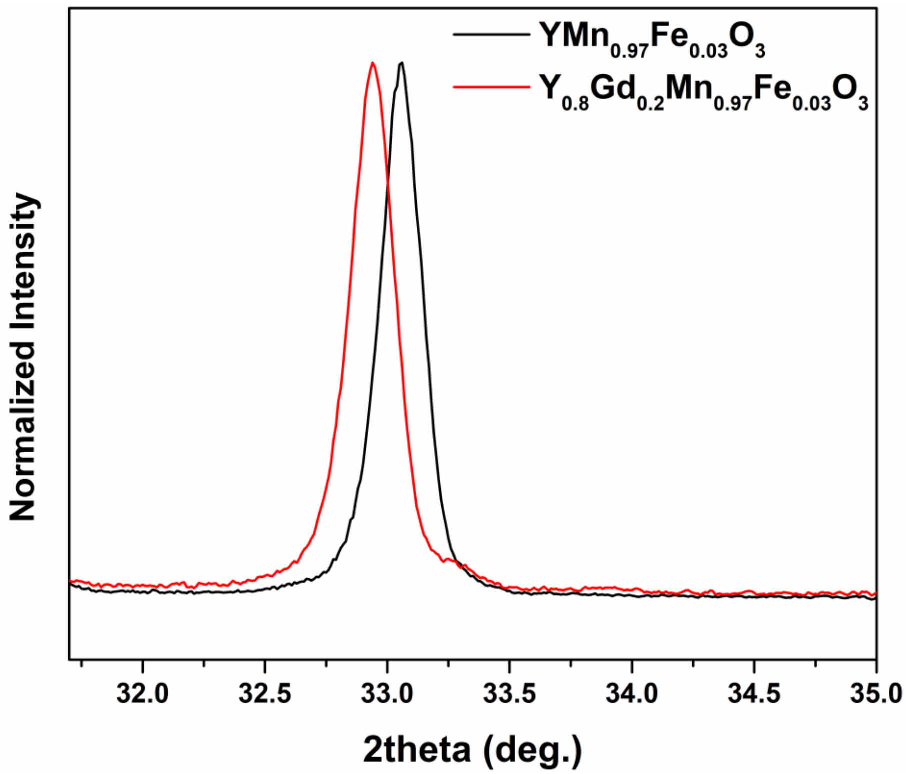

Figure S2. XRD patterns of $YMn_{0.97}Fe_{0.03}O_3$ and $Y_{0.8}Gd_{0.2}Mn_{0.97}Fe_{0.03}O_3$ samples demonstrating the peak shifting.

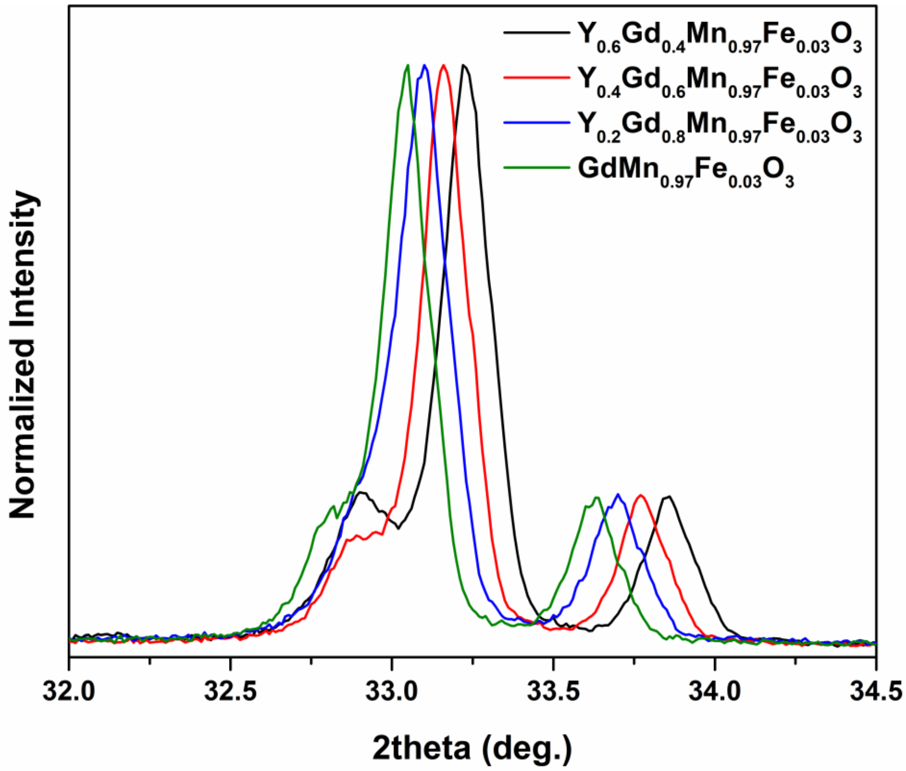

Figure S3. XRD patterns of $Y_xGd_{1-x}Mn_{0.97}Fe_{0.03}O_3$ samples with orthorhombic structure demonstrating the peak shifting.

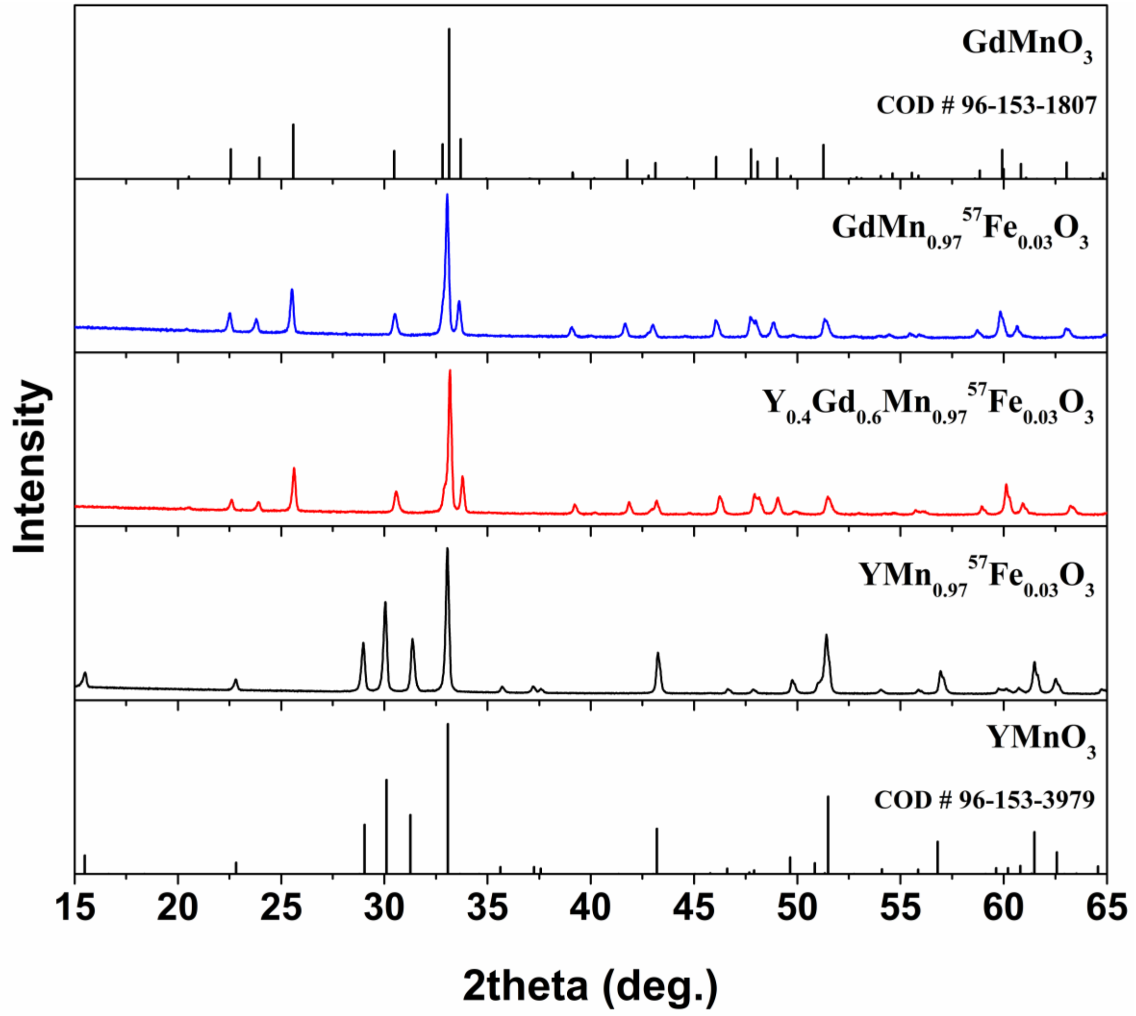

Figure S4. XRD patterns of $Y_xGd_{1-x}Mn_{0.97}{}^{57}Fe_{0.03}O_3$ samples.

Table S1. Cell parameters for $Y_xGd_{1-x}Mn_{0.97}{}^{57}Fe_{0.03}O_3$ samples.

Sample	Lattice parameters (Å)			
	a	*b*	*c*	*V*
$YMn_{0.97}{}^{57}Fe_{0.03}O_3$	6.1426(1)	6.1426(1)	11.3681(6)	371.47(2)
$Y_{0.4}Gd_{0.6}Mn_{0.97}{}^{57}Fe_{0.03}O_3$	5.8241(1)	7.4062(5)	5.2856(3)	227.99(5)
$GdMn_{0.97}{}^{57}Fe_{0.03}O_3$	5.8365(3)	7.4391(8)	5.3115(3)	230.62(1)
